# Butylated Hydroxytoluene (BHT) Inhibits PIN1 Exocytosis From BFA Compartments in Arabidopsis Roots

**DOI:** 10.3389/fpls.2020.00393

**Published:** 2020-04-08

**Authors:** Ivan A. Paponov, Vadym Budnyk, Martina Paponov, William Teale, Klaus Palme

**Affiliations:** ^1^Institute of Biology II/Botany, Faculty of Biology, Albert-Ludwigs-University of Freiburg, Freiburg, Germany; ^2^Division of Food Production and Society, Norwegian Institute of Bioeconomy Research (NIBIO), Ås, Norway; ^3^Centre of Biological Systems Analysis, Albert-Ludwigs-University of Freiburg, Freiburg, Germany; ^4^BIOSS Centre for Biological Signalling Studies, Albert-Ludwigs-University of Freiburg, Freiburg, Germany

**Keywords:** auxin, exocytosis, endocytosis, Brefeldin A, PIN-FORMED (PIN) proteins, antioxidant ionol, BHT, butylated hydroxytoluene

## Abstract

The activity of polarly localized PIN-FORMED (PIN) auxin efflux carriers contributes to the formation of auxin gradients which guide plant growth, development, and tropic responses. Both the localization and abundance of PIN proteins in the plasma membrane depend on the regulation of PIN trafficking through endocytosis and exocytosis and are influenced by many external and internal stimuli, such as reactive oxygen species, auxin transport inhibitors, flavonoids and plant hormones. Here, we investigated the regulation of endosomal PIN cycling by using a Brefeldin A (BFA) assay to study the effect of a phenolic antioxidant ionol, butylated hydroxytoluene (BHT), on the endocytosis and exocytosis of PIN1 and PIN2. BHT is one of the most widely used antioxidants in the food and feed industries, and as such is commonly released into the environment; however, the effect of BHT on plants remains poorly characterized. Preincubation of Arabidopsis seedlings with BHT before BFA treatment strongly enhanced the internalization of PIN1 into BFA compartments. After the simultaneous application of BHT and NAA, the NAA effect dominated PIN internalization suggesting the BHT effect occurred downstream to that of NAA. Washing seedlings with BHT after BFA treatment prevented the release of PIN1 from BFA compartments back to the plasma membrane, indicating that BHT application inhibited PIN1 exocytosis. Overall rates of PIN2 internalization were less pronounced than those of PIN1 in seedlings pre-incubated with BHT before BFA treatment, and PIN2 exocytosis was not inhibited by BHT, indicating a specific activity of BHT on PIN1 exocytosis. Comparison of BHT activity with other potential stimuli of PIN1 and PIN2 trafficking [e.g., H_2_O_2_ (ROS), salt stress, reduced glutathione (GSH), dithiothreitol (DTT), and flavonoids] showed that BHT has a new activity distinct from the activities of other regulators of PIN trafficking. The findings support BHT as a potentially interesting pharmacological tool for dissecting PIN trafficking and auxin transport.

## Introduction

Environmentally responsive plant growth requires the integration of diverse physiological responses. Many environmental stresses induce the formation of reactive oxygen species (ROS), the response to which often includes the biosynthesis of antioxidants that influence growth and development through their effect on plant hormones. Among these, auxins are prominently involved through their formation of instructive concentration gradients. These gradients are generated by PIN-FORMED (PIN) auxin efflux facilitator proteins, which may be asymmetrically localized to various sides of the cell, but always in the direction of auxin flux (Adamowski and Friml, 2015). This polar membrane localization of PINs and their abundance on the plasma membrane are established through the continuous circulation of PIN-containing endocytic vesicles from the plasma membrane to endosomes (endocytosis) and from the endosomes to the plasma membrane (exocytosis) (Geldner et al., 2001).

In Arabidopsis, the *PIN* gene family is represented by eight members whose protein products show a partially overlapping expression pattern that builds a dynamic network to allow differential regulation of auxin distribution among different cell types (Blilou et al., 2005; Paponov et al., 2005). Two *PIN* genes, *PIN1* and *PIN2*, were first identified and characterized based on their distinct phenotypes: *pin1* mutants formed a pin-like inflorescence stem without lateral organs (Okada et al., 1991; Gälweiler et al., 1998), while *pin2* mutants gave rise to agravitropic roots (Müller et al., 1998; Blilou et al., 2005). Despite the strong *pin1* phenotype observed in the shoot, a weak root phenotype with slight growth reduction was found (Blilou et al., 2005). However, the double mutant *pin1pin2* displayed stronger root phenotype when compared with either of the single mutants; this difference was explained by a significant degree of redundancy among members of the PIN family (Blilou et al., 2005; Paponov et al., 2005; Vieten et al., 2005). Therefore, the available evidence indicates that both PIN1 and PIN2 are important for root growth.

In the root, PIN1 and PIN2 are expressed mostly in non-overlapping domains: PIN1 is mostly expressed in stele and endodermis cells, while PIN2 is expressed in epidermis, cortex, and lateral root cap cells; PIN1 and PIN2 expression domains overlap in the cortex cells of the root apical meristem (Omelyanchuk et al., 2016). Nevertheless, PIN1 and PIN2 can show ectopic expression, expanding into the expression domain of the other in knock-out plants, partially replacing the missing function (Vieten et al., 2005). Importantly, the polar localization of the ectopically expressed PINs still reflects the localization of the missing PINs in the direction of expected polar auxin transport (Blilou et al., 2005; Paponov et al., 2005; Vieten et al., 2005).

Despite this apparent redundancy between PIN1 and PIN2, different processes regulate their expression and localization. Asymmetrical transport of PIN1 is regulated by a specific guanine-nucleotide-exchange factor that controls the ADP-ribosylation factor G-protein exchange factor (ARF-GEF), GNOM, by activation of an ADP-ribosylation factor (Steinmann et al., 1999). The localization of PIN1 proteins is thus regulated by GNOM (Geldner et al., 2003), but recycling of PIN2 is regulated by additional partially Brefeldin A (BFA)-sensitive ARF GEF(s) and by a retromer complex (Kleine-Vehn et al., 2008).

Different factors have been proposed to be involved in the regulation of cycles of PIN endocytosis and exocytosis. One of the first candidates for the regulation of PIN endocytosis was auxin itself, based on a positive feedback loop between auxin concentration and auxin transport (Paciorek et al., 2005) and a receptor role for ABP1 in this process (Robert et al., 2010). However, analysis of new *abp1* mutants (Gao et al., 2015) showed that the auxin response was independent of ABP1 (Paponov et al., 2019a). Most importantly, the natural auxin IAA only had a very weak influence over this process when compared to the artificial analog 1-NAA, the form of auxin most often used in experiments on the inhibition of endocytosis by auxin (Paponov et al., 2019b). The low activity of natural auxin with respect to PIN endocytosis emphasizes the importance of investigating other possible signals that may regulate the orchestration of the PIN network under different conditions. Indeed, several other signals have been identified including cytokinin (Marhavy et al., 2011), GOLVEN peptides (Whitford et al., 2012), and salicylic acid (Du et al., 2013).

In contrast to endocytosis, the process of exocytosis has received less attention. One of the most widely used chemical tool to inhibits exocytosis is a fungal toxin BFA which inhibit six of eight ARF-GEFs, essential regulators of vesicle trafficking (Anders and Jurgens, 2008). The earliest investigations of PIN1 trafficking indicated a role for two auxin transport inhibitors, NPA and TIBA, in the inhibition of both endocytosis and exocytosis (Geldner et al., 2001). Interestingly, flavonoids, which compete for NPA binding sites, also inhibited polar auxin transport, but they did not modulate PIN1 or PIN2 trafficking in wild-type Arabidopsis plants (Peer et al., 2004). More recently, the small molecule Endosidin2 was discovered to inhibit exocytosis and endosomal recycling in both plant and human cells and to enhance plant vacuolar trafficking (Zhang et al., 2016).

PIN trafficking may be regulated by ROS: ubiquitous and widely integrated stress-induced factors (Zwiewka et al., 2015; Yokawa et al., 2016). The discovery of the stimulatory effect of ROS on endocytosis and their inhibitory effect on exocytosis (Zwiewka et al., 2015) raises the question of whether ROS scavengers and antioxidants regulate PIN trafficking. Investigations with flavonoids showed that these naturally occurring antioxidants do not perturb PIN trafficking (Peer et al., 2004). Our recent investigation with a synthetic phenolic antioxidant, butylated hydroxytoluene (BHT) showed that BHT strongly stimulated PIN1 internalization in a BFA assay and had a weaker effect on PIN2 internalization (Paponov et al., 2019b). This enhanced PIN1 internalization might occur due to the stimulation of endocytosis and/or the inhibition of exocytosis. BHT, as one of the most widely used antioxidants in the food and feed industries, has been found as a pollutant in natural environments (Nieva-Echevarria et al., 2015). However, few investigations have examined the effect of BHT on plant cell physiology. As ROS affects PIN internalization, we hypothesize that BHT activity might modulate the level of ROS in cells. Here, we elucidated the effect of BHT on PIN1 and PIN2 endocytosis and exocytosis and compared the responses to BHT with those elicited by other antioxidants and ROS.

## Results

### Dose-Dependent Response of PIN Internalization to BHT

**FIGURE 1:**
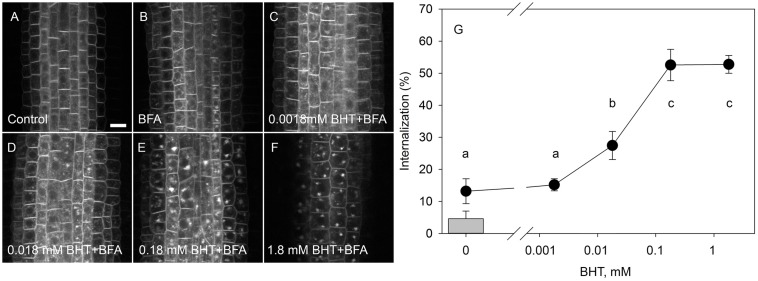
Dose-response of PIN1-GFP internalization in BHT. **(A)** PIN1-GFP localization under control conditions. **(B)** BFA (50 μM) induced PIN1-GFP internalization. Effect of 30 min pre-treatment with BHT supplied at 0.0018 mM **(C)**, 0.018 mM **(D)**, 0.18 mM **(E)**, and 1.8 mM **(F)** on PIN1-GFP internalization. Scale bars represent 10 μm. **(G)** Percentage of PIN1-GFP internalization extracted by quantification of the data represented in **(B–F)**. The column represents the level of PIN1-GFP internalization under control conditions **(A)**. Data are the means of 7–10 seedlings.

**FIGURE 2:**
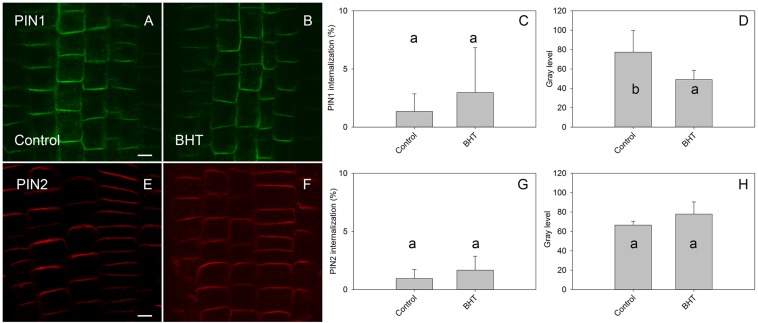
Effect of BHT on PIN localization. **(A,E)** PIN1 and PIN2 localization under control conditions. **(B,F)** Application of 200 μM BHT for 60 min in the absence of BFA did not change the localization of PIN1 and PIN2. Scale bars represent 5 μm. **(C,G)** Percentage of PIN1 and PIN2 internalization extracted by quantification of data presented in **(A,B,E,F)**. **(D,H)** Signal intensity of PIN1 and PIN2 at the plasma membrane in **(A,B,E,F)**. Data are the means of 6 seedlings; error bars represent SD. Means with the same letters are not statistically significant (*p* < 0.05).

To identify the minimal BHT concentration which saturated PIN1-GFP internalization, we performed a dose-dependent response internalization assay. The lowest BHT concentration which was sufficient to induce PIN1-GFP internalization was 0.018 mM, while the PIN1-GFP internalization response was saturated at 0.18 mM BHT after 30 min (Figure 1). Because a BHT concentration of 0.18 mM was sufficient to saturate PIN internalization, the following experiments were carried out at 0.2 mM to minimize any secondary effects of BHT that might be induced at higher concentrations. The application of BHT alone (in the absence of BFA) had no effect on the distribution of PIN1 and PIN2 between the plasma membrane and endomembrane compartments (Figure 2) indicating that the enhanced accumulation of PINs in BFA compartments was not due to a direct inhibition of exocytosis by BHT. The application of BHT alone decreased the intensity of the signal in the plasma membrane for PIN1 but not for PIN2, indicating a differential regulation of cellular trafficking routes by BHT. The reduction in PIN1 signal intensity might reflect an increased rate of endocytosis and/or a reduced rate of exocytosis. The absence of a BHT effect on PIN internalization in the absence of BFA led us to next test whether the application of BHT alone was sufficient to modulate the root growth response.

### Dose-Dependent Response of Root Growth to BHT

**FIGURE 3:**
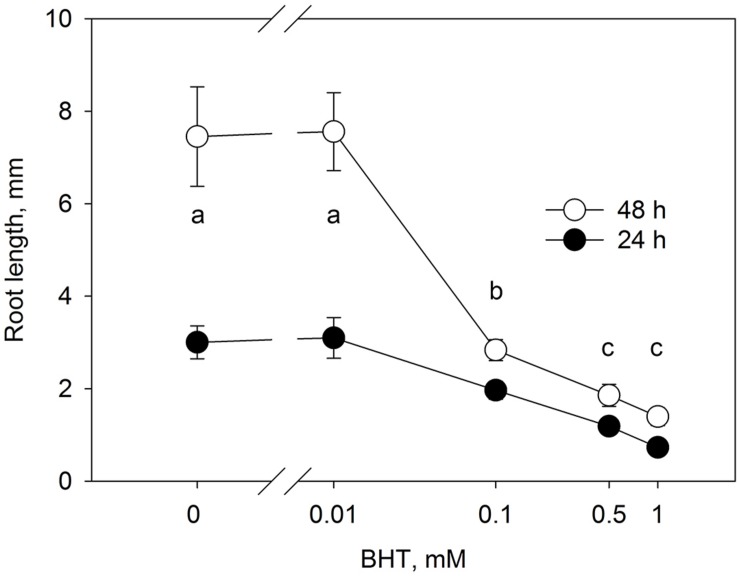
Dose-response of root growth in to BHT. Root growth after 24 and 48 h for Arabidopsis seedlings cultivated on media with different concentrations of BHT. Data are the means of 14–19 seedlings; error bars represent SD. Means with the same letters are not statistically significant (*p* < 0.05).

The root growth response to BHT (Figure 3) correlated closely with the dose-dependent response of PIN1-GFP internalization to BHT, indicating that, despite the qualitative maintenance of PIN localization and distribution, BHT might nevertheless affect the function of auxin transporters.

### BHT Affects PIN Internalization by Acting Downstream of NAA

In the BFA assay, BHT increased the rate of PIN internalization, whereas NAA inhibited it. Importantly, the application of different BFA concentrations was able to separate the processes which are involved in PIN internalization and PIN stability. For PIN2, a relatively low concentration of BFA (25 μM) preferentially inhibits recycling, whereas a high concentration (50 μM) also stops the targeting of vesicles to the vacuole for degradation (Kleine-Vehn et al., 2008). Thus, by understanding the effect on PIN internalization at low BFA concentrations we may accommodate the potential effects of BFA on the targeting of vesicles to the vacuole (Robert et al., 2010).

**FIGURE 4:**
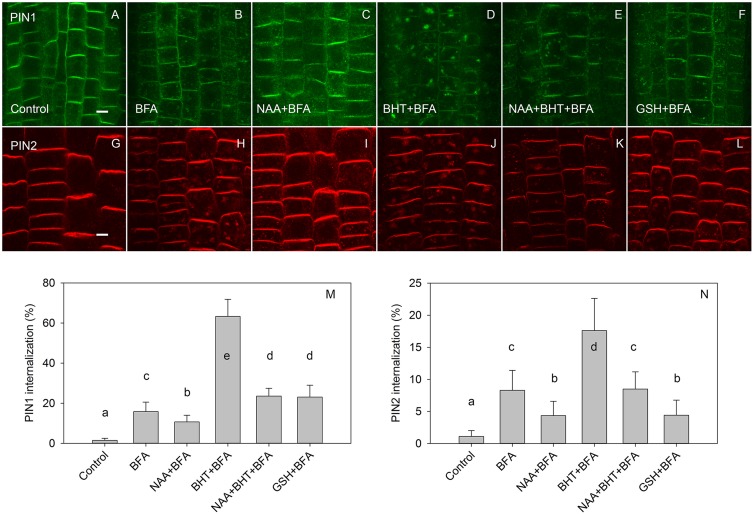
The effect of NAA, BHT, and GSH on PIN1 and PIN2 internalization. **(A,G)** PIN1 and PIN2 localization under control conditions. **(B,H)** BFA (25 μM) induced PIN1 and PIN2 internalization. **(C,I)** NAA (10 μM) inhibited BFA-induced PIN1 and PIN2 internalization. **(D,J)** BHT strongly increased internalization of PIN1, with a weaker effect on PIN2. **(E,K)** Simultaneous application of BHT and NAA weakly stimulated BFA-induced internalization for PIN1 but did not affect the internalization of PIN2. **(F,L)** GSH weakly increased the internalization of PIN1 and weakly decreased internalization of PIN2. Scale bars represent 5 μm. **(M,N)** Percentage of PIN1 and PIN2 internalization extracted by quantification of the data presented in **(A–L)**. Data are the means of 5–10 seedlings; error bars represent SD. Means with different letters are statistically significant (*p* < 0.05).

In agreement with experiments which used 50 μM BFA (Paponov et al., 2019b), the effect of BHT was stronger on PIN1 than it was on PIN2 internalization (Figure 4). However, at a low BFA concentration (25 μM), PIN2 was more sensitive to BHT, indicating that BHT could also be involved in PIN2 vacuolar targeting. The simultaneous application of BHT and NAA resulted in a level of PIN internalization more similar to that achieved after treatment with NAA alone than with BHT alone, suggesting that the BHT effects on PIN internalization are not due to an enhancement of PIN1 internalization but rather may be due to events occurring downstream of NAA. This observation is consistent with a hypothesis in which NAA inhibits PIN internalization, increasing the amount of PIN1 at the plasma membrane, and masking the downstream activity of BHT. The activity of BHT cannot be explained by changes which it induces in cellular redox status as the application of reduced glutathione did not lead to a similar effect on PIN1 and PIN2 internalization. Although PIN1 internalization was weakly increased, the opposite effect was observed for PIN2 (Figure 4). Taking into account that BHT acted downstream of NAA, we next investigated the effect of BHT on exocytosis.

### BHT Specifically Inhibits Exocytosis of PIN1 in a ROS-Independent Manner

**FIGURE 5:**
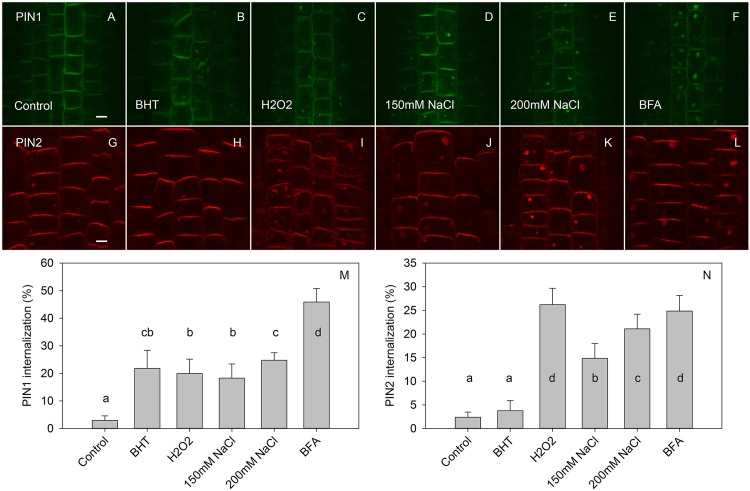
The effect of BHT and H_2_O_2_ on exocytosis of PIN1 and PIN2. **(A,G)** PIN1 and PIN2 immunolocalization after incubation for 45 min with BFA followed by 120 min of drug washout. **(B,H)** Washout with BHT strongly increased the PIN1 signal in BFA vesicles but had a weak effect on the PIN2 signal. **(C,I)** Washout with H_2_O_2_ prevented the disappearance of both PIN1 and PIN2 from the BFA-vesicle. **(D,E,J,K)** Washout with NaCl had a similar effect to that observed with H_2_O_2_. **(F,L)** Control treatment with continuous BFA treatment. Scale bars represent 5 μm. **(M,N)** Percentage of PIN1 and PIN2 internalization extracted by quantification of the data presented in **(A–L)**. Data are the means of 7–10 seedlings; error bars represent SD. Means with different letters are statistically significant (*p* < 0.05).

**FIGURE 6:**
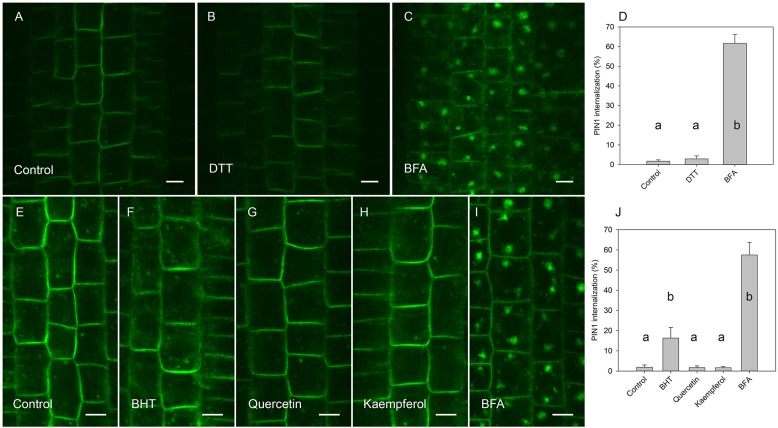
Effect of antioxidants on exocytosis of PIN1. **(A)** PIN1 immunolocalization after incubation for 45 min with BFA followed by 120 min of drug washout. **(B)** Washout with DTT did not increase PIN1 signal in BFA vesicles. **(C)** PIN1 immunolocalization by maintenance incubation of seedlings with BFA **(E)** PIN1 localization after incubation for 45 min with BFA followed by washing with half-strength Murashige and Skoog salts. **(F)** PIN1 localization after 120 min washing with 20 μM BHT, **(G)** with 20 μM quercetin, **(H)**, and with 20 μM kaempferol. **(I)** PIN1 localization by maintenance of incubation of seedlings in 50 μM BFA. Scale bars represent 5 μm. **(D)** Percentage of PIN1 internalization extracted by quantification of the data from the DTT experiment presented in **(A–C)**. **(J)** Percentage of PIN1 internalization extracted by quantification data from antioxidant experiment **(E–I)**. Data are the means of 6–8 seedlings; error bars represent SD. Differences between means with different letters are statistically significant.

By visualizing PINs after the washing out of BFA, we observed that the application of BHT stopped the release of PIN1 from BFA compartments to the plasma membrane, leading us to hypothesize that BHT inhibited exocytosis (Figure 5). However, BHT treatment did not inhibit the release of PIN2 from BFA compartments, suggesting that the observed BHT activity was specific to PIN1. It must be remembered here that BHT did not enhance the accumulation of PINs in the endosome compartments in the absence of BFA. BHT therefore does not appear to directly stop exocytosis. Instead, it is active in inhibiting exocytosis from the BFA compartments. We next tested whether the activity of BHT on PIN1 exocytosis was related to its being an antioxidant by comparing the effect of BHT on PIN exocytosis by applying either ROS (H_2_O_2_) or a ROS-induced stress (salt stress). One mM H_2_O_2_ inhibited the exocytosis of both PIN1 and PIN2 (Figure 5), indicating that the inhibition of PIN1 exocytosis by BHT might be due to its prooxidative rather than its oxidative properties. BHT did not, however, inhibit PIN2 exocytosis, therefore the activity of BHT cannot be explained solely by its antioxidant or prooxidant activity. Incubation with a reductant, DTT (Kovacik et al., 2009), did not affect exocytosis (Figure 6), further supporting the idea that the decrease in cellular ROS abundance is not sufficient to change PIN trafficking. Treatment with the natural flavonoids, quercetin and kaempferol, also did not affect the rate of PIN exocytosis (Figure 6), further supporting a unique type of inhibitory action of BHT on PIN1 exocytosis.

## Discussion

The PIN-dependent regulation of auxin distribution is one of the most important mechanisms for the initiation and maintenance of growth-coordinating auxin gradients. The abundance and localization of PINs on the plasma membrane depend on rates of both PIN endocytosis and exocytosis, as indicated by previous investigations that used specific drugs to perturb PIN activity (Doyle et al., 2015). New small molecules which can interfere with endocytosis and exocytosis are important tools for further investigations into the regulation of PIN trafficking (Huang et al., 2019). In the present work, we report that a phenolic ional antioxidant (BHT, E-321) is a promising chemical agent for studying PIN cycling, as it specifically inhibits the exocytosis of PIN1 but not PIN2.

### BHT Differs in Its Inhibition of PIN1 and PIN2 Exocytosis From BFA Compartments

The main finding of the present study is that BHT stops PIN1 exocytosis from BFA compartments without affecting PIN2 exocytosis. This regulation is ROS-independent. We cannot exclude the possibility that BHT also enhanced endocytosis, because a reduction in the signal of PIN1 on the plasma membrane and an increased PIN1 internalization during pre-incubation by BHT might occur due to a combination of inhibition of exocytosis and stimulation of endocytosis. Previous experiments with cycloheximide (CHX) showed that PIN1 was internalized in to BFA compartments and, upon withdrawal of BFA, PIN reappeared at the plasma membrane in a manner which was independent of protein synthesis (Geldner et al., 2001). Because the effect of BHT on PIN1 synthesis is unknown, we cannot exclude the possibility that a BHT-induced increase in PIN1 internalization was due to increased *de novo* PIN1 biosynthesis. However, the fact that the PIN1 signal in the cell did not increase after BHT treatment (Figure 2), and that the PIN1 level was reduced on the plasma membrane, suggests that the main mechanism of BHT action is related to inhibition of exocytosis. Taken together, the evidence presented here indicates that BHT might directly affect PIN1 trafficking by interacting with as yet unidentified targets, rather than acting through the regulation of ROS abundance.

BHT is one of the most widely used antioxidants in the food industry and has been extensively investigated in animal systems and in terms of human health (Nieva-Echevarria et al., 2015); however, plant responses to BHT have received less attention. Nevertheless, several interesting and unusual activities of BHT have been discovered in plants, such as an induction of shoot branching (Grochowska and Buta, 1985). At the cellular level, BHT induces large structural changes in the Golgi apparatus and the endoplasmic reticulum (Bakeeva et al., 2001), suggesting that BHT not only blocks exocytosis of PIN1 but also interferes with other cell functions.

We suggest three alternative hypotheses to explain the modulation of PIN trafficking by BHT observed in our investigation. The first hypothesis is that BHT acts as an antioxidant and indirectly regulates PIN trafficking by ROS scavenging. The second hypothesis is that BHT acts as a prooxidant to produce ROS that then affect PIN trafficking. The third hypothesis is that BHT acts directly on some as yet unidentified molecular targets to modulate PIN trafficking.

### BHT Action Differs From That of Other Antioxidants

If our first hypothesis—that BHT acts as a ROS scavenger in modulating PIN trafficking—were correct, we would expect that BHT effects counteract ROS effects. However, BHT and ROS both inhibited PIN1 exocytosis; they also had unrelated actions with respect to PIN2 trafficking (Figure 5), BHT did not change PIN2 exocytosis, whereas H_2_O_2_ inhibited exocytosis (Zwiewka et al., 2015).

A different pattern of activity for PIN1 and PIN2 trafficking was also indicated by our experiment with the reduced form of glutathione (GSH) (Figure 4) and the reducing agent DTT (Figure 6), both of which are known to reduce ROS concentration in plants (Kovacik et al., 2009; Yin et al., 2017). These results indicated that the specific effect of BHT on PIN trafficking cannot be explained exclusively by the modulation of ROS concentration. Interestingly, GSH and BHT showed different activity patterns, differentially modulating PIN1 and PIN2 internalization. GSH enhanced PIN1 internalization, possibly by stimulation of PIN1 endocytosis and/or inhibition of exocytosis. This GSH activity cannot be explained by the antioxidant properties of GSH because ROS showed the same activity on PIN1 internalization. However, GSH acted as an antioxidant, decreasing PIN2 internalization in a reverse response to that induced by ROS (Zwiewka et al., 2015; Figure 5). The antioxidant effects of GSH on PIN2 internalization but not on PIN1 internalization might be related to the localization of PIN2 in the outermost layers of the roots (epidermis and cortex), as these layers are more sensitive to external stimuli and exogenous GSH application. This difference in GSH responses further supports the importance of redox status in the regulation of polar auxin transport. However, the transcriptional regulation of *PIN* expression seems to be the main mechanism regulating PIN activity under oxidative conditions in Arabidopsis roots (Koprivova et al., 2010; Yu et al., 2013).

### BHT Does Not Act as a Pro-oxidant

The second hypothesis—that BHT acts as a pro-oxidant that affects PIN trafficking—is supported by the observation that BHT and H_2_O_2_ act in a similar manner on PIN1 exocytosis (Figure 5). Pro-oxidant activity is possible for BHT since some antioxidants do behave as pro-oxidants under certain circumstances (Valko et al., 2004). Indeed, BHT can interact with oxygen in aqueous media (aerobic conditions) to generate O_2_^*–^ (superoxide) (Smirnova et al., 2002), whereas inside the seedlings, BHT acts as a ROS scavenger (Smirnova et al., 2002).

Based on the potential for the production of ROS in an aqueous medium at the root surface and the fact that ROS inhibit exocytosis (Zwiewka et al., 2015 and as shown in Figure 5), this hypothesis predicts that BHT would inhibit the exocytosis of PIN2, which is expressed in the epidermis, but not of PIN1, which is expressed in the endodermis and central cylinder. However, the observed BHT response was opposite to this expectation: BHT inhibited PIN1 exocytosis and had no effect on PIN2 exocytosis (Figure 5). Thus, the different patterns of exocytosis of PIN1 and PIN2 in response to BHT and H_2_O_2_ do not support the idea that the activity of BHT on PIN trafficking is not related to its potential prooxidant activity.

Interestingly, hydrogen peroxide inhibits exocytosis but also stimulates endocytosis, as observed by its effects in the absence of BFA (Zwiewka et al., 2015). By contrast, BHT did not enhance endocytosis, as the application of BHT alone did not affect PIN internalization (Figure 2). This difference between H_2_O_2_ and BHT activities provide further support for the conclusion that the mode of BHT action cannot be explained by its pro-oxidant activity.

### BHT Action Differs From That of the Auxin Transport Inhibitors NPA and TIBA

The inhibitory effect of BHT on PIN1 exocytosis resembles that of the auxin transport inhibitors NPA and TIBA. Early studies on the PIN trafficking machinery showed that TIBA and NPA blocked the transfer of PIN1 from the BFA compartments to the membrane that occurred during cell washing after BFA treatment (Geldner et al., 2001). However, the pattern of BHT activity on PIN trafficking differs from the activity of the transport inhibitors, because TIBA and NPA inhibit both exocytosis and endocytosis (Geldner et al., 2001). By contrast, pre-treatment with BHT stimulated PIN internalization, indicating that BHT did not inhibit endocytosis (Paponov et al., 2019b; Figures 1, 4).

The activity of auxin transport inhibitors on auxin transport activity has been attributed to their binding to the multi-drug resistant ABCB type transporters, a plasma membrane aminopeptidase, and other proteins (including PINs themselves) (Teale and Palme, 2018). The sensitivity to NPA is lower for PIN trafficking than that for polar auxin transport, indicating that inhibition of auxin transport is not due to blocking of PIN trafficking (Petrasek et al., 2003). By contrast, the dose responses of PIN internalization and root growth to BHT treatment (Figure 3) showed similar sensitivities, indicating that the inhibition of root growth by BHT might be due to the blocking of PIN trafficking. The identified differences in activities between NPA/TIBA and BHT indicate that the mechanism of action of BHT is likely to differ from that of auxin transport inhibitors.

### Differences Between BHT and Flavonoid Action

In contrast to BHT, flavonoids did inhibit PIN1 exocytosis (Figure 6), indicating that the mechanism of BHT action is also different from that of these naturally occurring antioxidants. The inability of flavonoids to inhibit PIN1 exocytosis agrees with previously published data for wild-type plants (Peer et al., 2004). This action of flavonoids differs from that of auxin transport inhibitors, although flavonoids and NPA share binding sites on NPA-interacting proteins (Jacobs and Rubery, 1988). Interestingly, although flavonoids do not inhibit PIN1 exocytosis in wild-type Arabidopsis, flavonoids inhibit PIN1 exocytosis in the flavonoid-deficient mutant, *tt4* (Peer et al., 2004). In contrast to PIN1 trafficking, PIN2 trafficking was not affect by flavonoids in the *tt4* mutant (Peer et al., 2004), further supporting different mechanisms for the regulation of PIN1 and PIN2.

### Differences Between BHT and BFA

The application of BHT alone did not induce PIN internalization, supporting the existence of different targets of BHT and BFA actions. Inhibition of PIN exocytosis in BFA wash-out experiments indicates BHT interference with cell recovery after the removal of BFA from cells.

### Differences Between BHT and Endosidin2

Endosidin2 has also shown an ability to inhibit the recovery of PIN trafficking from the BFA compartment to the plasma membrane (Zhang et al., 2016). However, this drug inhibits PIN2 recovery from the BFA compartment, and endosidin2 application also reduced PIN2 abundance on the membrane. Our finding that BHT did not affect PIN2 recovery from BFA compartments and did not reduce the abundance of PIN2 on the membrane indicates that endosidin2 activity differs from that of BHT.

Based on our BFA assay, we identify BHT as a chemical which is able to inhibit PIN1 exocytosis. This activity suggests that BHT might be a promising pharmacological tool for the investigation of PIN trafficking regulation under different genetic and environmental conditions and can be potentially used as a tool to study membrane trafficking in other organisms.

## Materials and Methods

*Arabidopsis thaliana* (L.) Heynh. Columbia (Col-0) and PIN1:PIN1:GFP (Benkova et al., 2003) seeds were surface sterilized for 10 min in 80% ethanol, 5% w/v calcium hypochlorite and 0.1% Triton X-100. After three washes in 80% ethanol and one in 100% ethanol, seeds were left to dry under sterile conditions. Seeds were sown on plates containing *Arabidopsis* medium [AM: half-strength Murashige and Skoog (MS) salts and 1% sucrose, pH 5.7] and 15 g l^–1^ agar-agar (Merck). After stratification overnight at 4°C in darkness, plates were transferred to a growth chamber (16 h light/8 h darkness, 21°C, 100 μM m^–2^ s^–1^) for seed germination and maintained in a vertical position. Experiments were performed on 4-day-old seedlings in 24-well cell-culture plates in liquid AM medium. For the evaluation of BFA-induced PIN internalization, Arabidopsis seedlings were pre-treated for 30 min in AM containing 10 μM 1-NAA, 200 μM BHT, or 1mM GSH. Pre-treatments were followed by 45 min of concomitant treatment with chemicals and 25 μM or 50 μM BFA. BFA was initially dissolved in dimethylsulfoxide (DMSO) at 50 mM. Control treatments contained an equal amount of DMSO. For the evaluation of the direct effect of BHT on PIN endocytosis, seedlings were treated for 30 min in AM containing 200 μM BHT. For the evaluation of PIN exocytosis from BFA-bodies to the plasma membrane (exocytosis), 4-day-old Arabidopsis seedlings were pretreated with 50 μM BFA (dissolved in the liquid 0.5 MS salt medium) for 45 min. BFA was removed by washing for 2 h with 0.5 MS medium containing appropriate chemicals (1 mM H_2_O_2_, 1 mM DTT, 200 μM BHT, 50 μM BFA, 150 mM NaCl, or 200 mM NaCl).

Immunolocalization of Arabidopsis roots was carried out as described previously (Friml et al., 2003). Rabbit anti-PIN1 (Gälweiler et al., 1998) and guinea pig anti-PIN2 (Tromas et al., 2009) were diluted 1:500. Alexa 488- and Alexa 555-conjugated anti-rabbit (for PIN1) and anti-guinea pig (for PIN2) secondary antibodies were diluted 1:400. Solutions were changed during the immunolocalization procedures were changed using a pipetting robot (*Insitu* Pro; Intavis).

Confocal images were taken using a Zeiss LSM 510 NLO confocal laser scanning microscope. Alexa Fluor 488 and GFP were excited with a 488 nm argon laser line in combination with a 500–550 band-pass filter. Alexa Fluor 555 was excited with a helium-neon 543 nm laser (HeNe laser) in conjunction with a 575-long-pass filter. The confocal microscopy images were quantitatively analyzed using Imaris 7.5.6 software (Bitplane AG). The fluorescence signal was detected using the “create surface” tool, and the fluorescence signal was calculated at the plasma membrane and in the BFA bodies. The level of signal internalization (the signal in the BFA bodies) was calculated as the ratio between intensity of the intracellular fluorescence signal and the intensity of the total fluorescence signal and expressed as a percentage. For every root, the estimation of the level of PIN internalization was based on 20–32 and 10–18 cells for PIN1 and PIN2, respectively. Quantification of PIN1 and PIN2 signal in plasma membrane was performed along defined linear region of interest (ROI) drawn crossing cells using ImageJ. We used 5–10 roots for every treatment. Averages for every root were used for statistical analysis.

For root length measurements Arabidopsis seedlings were grown on vertically oriented plates in a growth chamber under the standard conditions described above. Seedlings were grown for three days on control medium and then transferred onto new plates containing the same medium with a reduced percentage of agar (0.8% instead of 1.5%) and supplemented with BHT at 0, 10, 100, 500, or 1000 μM. The seedlings were then grown for another two days and the length of the main root was measured by scanning at 24 h and 48 h after seedling transfer and using ImageJ software (Wayne Rasband, National Institute of Health, United States).

Data were statistically analyzed by analysis of variance (one-way ANOVA). When significant treatment effects were identified by ANOVA, Fisher’s protected LSD test was used to compare the individual means (Statistica for Windows, version 13).

## Data Availability Statement

All datasets generated for this study are included in the article/supplementary material.

## Author Contributions

IP and KP conceived the project. IP, MP, and WT wrote the manuscript. VB performed the experiments and analyzed the data.

## Conflict of Interest

The authors declare that the research was conducted in the absence of any commercial or financial relationships that could be construed as a potential conflict of interest.

## Abbreviations

BFA: brefeldin A
BHT: butylated hydroxytoluene
GSH: reduced glutathione
DTT: dithiothreitol
1-NAA: 1-naphthaleneacetic acid.
